# The triphenylmethane dye brilliant blue G is only moderately effective at inhibiting amyloid formation by human amylin or at disaggregating amylin amyloid fibrils, but interferes with amyloid assays; Implications for inhibitor design

**DOI:** 10.1371/journal.pone.0219130

**Published:** 2019-08-12

**Authors:** Rehana Akter, Alexander Zhyvoloup, Bingqian Zheng, Surita R. Bhatia, Daniel P. Raleigh

**Affiliations:** 1 Department of Chemistry, Stony Brook University, Stony Brook, NY, United States of America; 2 Institute of Structural and Molecular Biology, University College London, Gower Street, London, United Kingdom; Nathan S Kline Institute, UNITED STATES

## Abstract

The development of inhibitors of islet amyloid formation is important as pancreatic amyloid deposition contributes to type-2 diabetes and islet transplant failure. The Alzheimer’s Aβ peptide and human amylin (h-amylin), the polypeptide responsible for amyloid formation in type-2 diabetes, share common physio-chemical features and some inhibitors of Aβ also inhibit amyloid formation by h-amylin and vice versa. Thus, a popular and potentially useful strategy to find lead compounds for anti-amylin amyloid agents is to examine compounds that have effects on Aβ amyloid formation. The triphenylmethane dye, brilliant blue G (BBG, Sodium;3-[[4-[(E)-[4-(4-ethoxyanilino)phenyl]-[4-[ethyl-[(3-sulfonatophenyl)methyl]azaniumylidene]-2-methylcyclohexa-2,5-dien-1-ylidene]methyl]-N-ethyl-3-methylanilino]methyl]benzenesulfonate) has been shown to modulate Aβ amyloid formation and inhibit Aβ induced toxicity. However, the effects of BBG on h-amylin have not been examined, although other triphenylmethane derivatives inhibit h-amylin amyloid formation. The compound has only a modest impact on h-amylin amyloid formation unless it is added in significant excess. BBG also remodels preformed h-amylin amyloid fibrils if added in excess, however BBG has no significant effect on h-amylin induced toxicity towards cultured β-cells or cultured CHO-T cells except at high concentrations. BBG is shown to interfere with standard thioflavin-T assays of h-amylin amyloid formation and disaggregation, highlighting the difficulty of interpreting such experiments in the absence of other measurements. BBG also interferes with ANS based assays of h-amylin amyloid formation. The work highlights the differences between inhibition of Aβ and h-amylin amyloid formation, illustrates the limitation of using Aβ inhibitors as leads for h-amylin amyloid inhibitors, and reinforces the difficulties in interpreting dye binding assays of amyloid formation.

## Introduction

**Fig 1:**
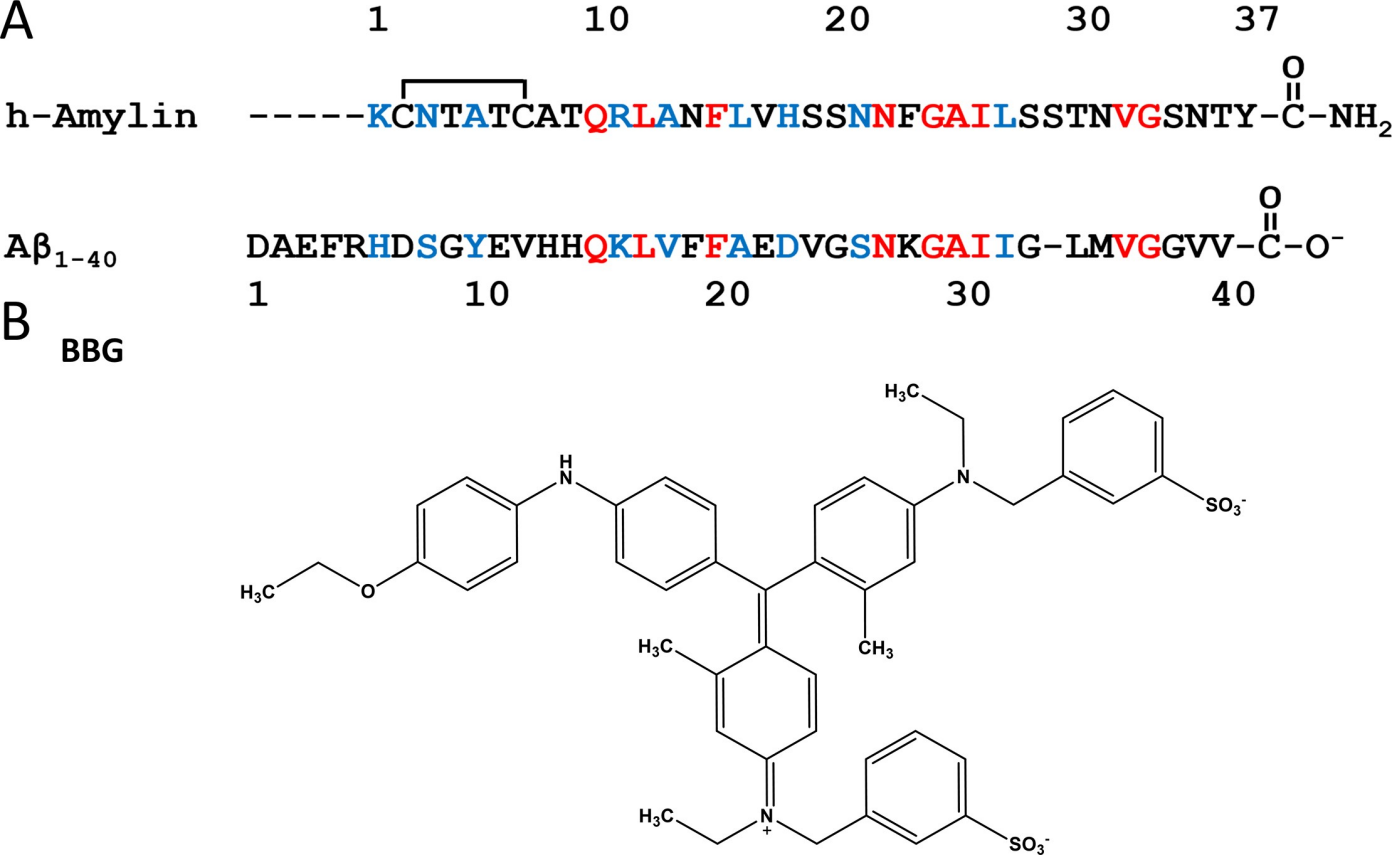
(A) Alignment of the primary sequence of h-amylin and Aβ_1–40_. The sequence alignment was performed using the program ALIGN (http://www.ch.embnet.org/software/LALIGN_form.html). Red and blue represent sequence identity and sequence similarity respectively. h-Amylin contains a conserved disulfide between Cys-2 and Cys-7 and an amidated C-terminus. (B) Structure of brilliant blue G (BBG).

The pathological aggregation of proteins to form amyloid or other aggregates contributes to a range of devastating human diseases including Alzheimer’s disease, Huntington’s disease, Parkinson’s disease and type-2 diabetes (T2D) [1–3]. In T2D, the normally soluble pancreatic polypeptide amylin forms extracellular amyloid deposits in the islets of Langerhans [4–6]. The process of islet amyloid formation contributes to β-cell dysfunction and to the loss of β-cell mass in T2D [6–13]. Islet amyloidosis also contributes significantly to islet graft failure and is an important factor limiting islet transplantation [14, 15]. There are no clinically approved inhibitors of islet amyloidosis despite their biomedical relevance. One strategy for developing lead compounds to inhibit amyloid formation and toxicity by a particular polypeptide or protein is to examine molecules that inhibit amyloid formation by other polypeptides. In the context of amylin, the suitable polypeptide is the Aβ peptide of Alzheimer’s disease. Human amylin (h-amylin) shares common features with Aβ and some inhibitors of Aβ amyloid formation inhibit amyloid formation by h-amylin and vice-versa [16–20]. In addition, it has been proposed that toxic oligomers formed by a range of amyloidogenic proteins share common features, suggesting that there may be compounds which inhibit multiple forms of amyloid [21–24]. Aβ and h-amylin have 25% sequence identity and approximately 50% similarity, with the higher identity and similarity in regions that have been proposed to be critical for amyloid formation (Fig 1). Both polypeptides are natively unfolded in their monomeric states. h-Amylin and Aβ interact *in vitro* and association have been demonstrated in plasma [7, 17, 18, 25]. Peptide mapping studies have shown that regions of h-amylin that are important for self-association *in vitro* are also “hot spots” for h-amylin Aβ hetero-interactions [18]. Aβ can seed amyloid formation by h-amylin in a mouse model and h-amylin has been reported in brain plaques in Alzheimer’s disease while Aβ has been reported to form pancreatic deposits in T2D [19, 26, 27]. These observations indicate that studies of known Aβ inhibitors are a potentially promising strategy for finding h-amylin amyloid inhibitors.

The triphenylmethane based compound brilliant blue G (BBG, Sodium;3-[[4-[(E)-[4-(4-ethoxyanilino)phenyl]-[4-[ethyl-[(3-sulfonatophenyl)methyl]azaniumylidene]-2-methylcyclohexa-2,5-dien-1-ylidene]methyl]-N-ethyl-3-methylanilino]methyl]benzenesulfonate) has been shown to: (1) inhibit Aβ induced toxicity towards cultured cells, (2) cross the blood brain barrier and (3) modulate amyloid formation by Aβ [28–30]. Given that other triphenylmethane derivatives are effective inhibitors of h-amylin amyloid formation and given the effects of BBG on Aβ, it is worthwhile examining the effect of the compound on h-amylin [31, 32]. Here we show that BBG has only modest effects on h-amylin amyloid formation and h-amylin induced toxicity towards cultured cells unless added in large excess, but interferes with the widely employed thioflavin-T dye based assays of amyloid formation and disaggregation. We also show that BBG infers with 1-anilinonaphthalene-8-sulphonic acid (ANS) assays of h-amylin amyloid formation. The implications for inhibitor design are discussed.

## Materials and methods

### Peptide synthesis and purification

h-Amylin was synthesized on a 0.1 mmol scale using standard Fmoc (9-fluorenyl methoxycarbonyl) microwave assisted solid phase peptide synthesis methods, with a CEM Liberty automated microwave peptide synthesizer. Fmoc-PAL-PEG-PS resin was used to obtain an amidated C-terminus. Fmoc protected pseudoproline (Oxazolidine) dipeptide derivatives were used to facilitate synthesis as previously described [33, 34]. All solvents were ACS grade. Fmoc-PAL-PEG-PS resin was purchased from Applied Biosystems. Fmoc protected amino acids and all other reagents were purchased from AAPPTec, Novabiochem, Sigma-Aldrich, VWR and Fisher Scientific. Standard reaction cycles were used. The first amino acid attached to the resin, pseudoproline dipeptide derivatives and all β-branched amino acids were double coupled. The peptide was cleaved from the resin and side chains protecting groups were removed using standard TFA (trifluoroacetic acid) methods. The crude peptide was dissolved in 100% DMSO at 10 mg/ml to promote intramolecular disulfide bond formation and allowed to stand at least for 72 hours at room temperature. The oxidized peptide was purified via reversed- phase HPLC using a C18 2.5 X 22.5 cm column (from Higgins Analytical). HCl was used as the counter ion. The dried peptide was dissolved in HFIP (1, 1, 1, 3, 3, 3-Hexafluoro-2-propanol) after the first purification to remove residual scavengers, and re-purified using reversed-phase HPLC. The purity of the peptide was checked by analytical HPLC using a C18 column and a single peak was detected. The molecular weight of the purified peptide was confirmed by mass spectrometry (h-amylin expected, 3903.30; observed 3903.90).

### Sample preparation

h-Amylin was dissolved in 100% HFIP to prepare a 0.5 mM stock solution, and aliquots were filtered through a 0.45 μm syringe-driven filter. The concentration of the samples was determined by measuring the absorbance at 280 nm. Aliquots were freeze dried to remove HFIP. BBG was obtained from Sigma-Aldrich (product no. B0770). A 1 mM BBG stock solution was prepared in 20 mM Tris-HCl with 140 mM KCl at pH 7.4.

### Liquid chromatography-mass spectrometry

LC-MS experiments were performed using an Agilent 1260 HPLC instrument with a Kinetex F5 column and an Agilent G6224A TOF mass spectrometer.

### Thioflavin-T fluorescence assays

Thioflavin-T fluorescence was measured using an excitation wavelength of 450 nm and an emission wavelength of 485 nm with a Spectramax Gemini EM plate reader. Samples were incubated in Corning 96-well non-binding surface black plates with lids and plates sealed with polyethylene sealing tape. Dry peptide samples were dissolved in Tris-HCl with 140 mM KCl buffer containing BBG and 32 μM thioflavin-T right before the kinetics assays. BBG was added at equimolar, 5-fold and 10-fold excess to peptide. The final concentration of the h-amylin was 16 μM. Experiments were conducted at 25°C, pH 7.4. without agitation.

Fibril disaggregation assays were performed by adding an equimolar and 10-fold excess of BBG to already formed h-amylin amyloid fibrils at 20 h. Experimental conditions were the same as used for the BBG inhibition assays.

### ANS fluorescence assays

1-anilinonaphthalene-8-sulphonic acid (ANS) was purchased from Acros Organics. Sample preparation for ANS assays and experimental conditions for the assays were similar to those used for the thioflavin T assays as described above, except that 4 μM ANS dye was used for the kinetic assays. ANS experiments were performed using a 370 nm excitation wavelength and a 500 nm emission wavelength.

### Sedimentation assays

16 μM dry peptide samples were dissolved in Tris-HCl with 140 mM KCl buffer containing 160 μM BBG at pH 7.4 or 16 μM BBG at pH 7.4 for 46 h. 16 μM and Fibril growth kinetics were monitored with a separate peptide sample with 32 μM thioflavin-T. Once fibrils were formed, samples containing BBG with and without h-amylin were centrifuged at 17,500 g for an hour and the absorbance of BBG in the supernatant was measured at 584 nm.

### Transmission electron microscopy

TEM images were recorded using an FEI Bio TwinG^2^ Transmission Electron Microscope at the Central Microscopy Center at Stony Brook University (Stony Brook, NY). 15 μL aliquots were removed from the kinetics experiments at designated times and blotted on a carbon-coated formvar 300-mesh copper grid for 1 min and then negatively stained with 2% uranyl acetate for 1 min. Images were taken at a 68,000X magnification and 100 nm under focus.

### Small angle X-ray scattering

Small angle X-ray scattering was conducted at Brookhaven National Laboratory National Synchrotron Light Source II (NSLS-II) on the Life Science X-ray Scattering (LiX) beamline, 16-ID. SAXS data was collected for a buffer blank and samples containing BBG at concentration of 0.5 mM and 1 mM. Data reduction and normalization were performed using pyXS [35]. The correlation length of BBG was estimated by the fitting the reduced data to the correlation length model developed by Hammouda and coworkers [36]. The experimental radius of gyration (*R*_*g*_) was calculated based on fit parameters (S2 Fig). The molecular dimensions of BBG were estimated for comparing to the experimental *R*_*g*_ using Chem3D. *R*_*g*_ was approximated using these estimates and the on line resource: https://www.staff.tugraz.at/manfred.kriechbaum/xitami/java/rgcalc.html.

### Atomic force microscopy

The AFM measurements were conducted using an Asylum MFP-3D AFM at the Center for Functional Nanomaterials (CFN), Brookhaven National Laboratory. 30 μL of the sample was deposited on a freshly cleaved mica disc. After 30 min of incubation at room temperature, excess sample was removed by rinsing with 1 mL of D.D.I. water and dried under a gentle stream of N_2_. The samples were imaged in tapping mode with silicone probes of an elastic modulus of 40 N/m and a frequency of 320 kHz.

### h-Amylin cytotoxicity assays and BBG cell protection assays

INS-1 cells were purchased from AddexBio and cultivated in optimized RPMI-1640 (AddexBio, #C0004-02) supplied with 10% ultra-low IgG FBS (Gibco, #16250078). Alamar Blue (Generon, #MBS238967), CellTiter-Glo 2.0 (Promega, #G9242) and CellTox Green (Promega, # G8741) assays were used to evaluate the cytotoxicity of h-amylin to INS-1 cells and the protective properties of BBG. Alamar Blue assays were used to examine the effect of h-amylin and BBG on CHO-T cells. Evaluation of the cytotoxicity of h-amylin towards INS-1 cells was performed as follows. The cells were seeded at ~50% confluence on a 96-well half-area clear bottom white plates (Greiner, #675083) and incubated in humidified 5% CO_2_-incubator at 37°C for 36 hours. Serial dilutions of the peptides were freshly prepared before use from the lyophilized aliquots. Cultured cells were exposed to the peptide diluted in the fresh complete medium for further 24 hours.

For the BBG cell protection experiments the plated INS-1 cells were seeded and conditioned as above. The culture medium was replaced by freshly prepared 2X serial dilutions of BBG in complete culture medium and the plates were incubated in a humidified, 5% CO_2_*-*incubator at 37°C for 30 min. Then an equal volume of a freshly prepared 80 μ M h-amylin in complete medium was added and the plates were further incubated for 24 hours. The final concentration of h-amylin was 40 μM.

All cell viability assays were conducted according to the *manufacturer*'s protocols. Briefly, an equal volume of 20% Alamar Blue reagent in complete medium was added to the treated cells and the plates were further incubated at 37°C for ~1 h. Fluorescence of the Alamar Blue reduction product was measured using excitation/emission wavelengths of 550 nm and 590 nm respectively. For the CellTiter-Glo 2.0 assays, the cultured plates were cooled to room temperature and an equal volume of the assay reagent was added to the treated cells. The plates were vigorously (700 rpm) shaken for 1 min and luminescence intensity was measured on a plate reader. For the CellTox Green assays, the cells were exposed simultaneously to h-amylin and the assay dye for 24 hours before the fluorescence intensity was measured using excitation at 480 nm and emission at 525 nm. Statistical analysis and calculation of EC_50_ values were completed using Graph Pad Prism 5.

## Results

### Brilliant blue G interferes with thioflavin-T and ANS based assays of h-amylin amyloid formation

The primary sequence of h-amylin and Aβ are displayed in (Fig 1). h-Amylin is a 37 residue polypeptide with a disulfide bridge connecting Cys-2 and Cys-7 and an amidated C-terminus. The polypeptide contains no acidic residues and the amidated C-terminus ensures that h-amylin will have a net positive charge at all physiologically relevant pH values. Aβ is commonly found as two isoforms Aβ_1–40_ and Aβ_1–42_ differing at the C-terminus.

We first tested the purity and stability of commercially available BBG in buffer using liquid chromatography- mass spectrometry (LC-MS). The samples appear to be greater than 96.5% pure as judged by absorbance monitored LC. A small amount of impurity is observed at the 2 to 3% level as judged by the absorbance at 610 nm with a mass which corresponds to a loss of 170 Da (S1 Fig). This is consistent with loss of a sulfonated aromatic group. The level of the apparent impurity appears higher, >10%, if the total ion count is monitored. We tested two independent samples of BBG and obtained identical results in both cases. No change in the LC trace was observed after 24 hours incubation of the compound in buffer, indicating, as expected, that the compound is stable (S1 Fig).

A number of apparent small molecule inhibitors of amyloid formation can themselves aggregate in isolation, and the resulting aggregates can sequester amyloidogenic proteins. Such so-called colloidal inhibitors are not useful since they can interact non-specifically with a wide range of proteins. Thus, we first examined the tendency of BBG to self-associate under the conditions of our measurements. The strong absorbance of BBG prevented DLS measurements as the frequency of the laser light scattering source overlaps with a region of significant absorbance of BBG. We thus conducted small angle X-ray scattering studies (SAXS) of BBG at 0.5 mM and 1 mM concentrations. These concentrations are higher than those used for most of the inhibition studies, but are required to obtain an adequate signal to noise. Fitting the scattering curves yields an estimated experimental *R*_*g*_ of 20 Å (S2 Fig). The molecular dimensions of BBG were estimated using Chem3D. The long axis of the molecule was found to 19.5 Å and the other two 13.6 Å and 15.7 Å. The expected *R*_*g*_, estimated using these values, is on the order of 8 to 9 Å. The experimental value of *R*_*g*_ is consistent with the absence of significant amounts of high order aggregates of the compound, but indicates some tendency to form low order complexes such as dimers and trimers at the concentrations tested. The lack of a sharp increase in the scattering intensity at low *q* is also consistent with the lack of large aggregates. The signal to noise of the scattering curve prevents a more detailed analysis of the results.

We examined the ability of an equimolar amount of BBG to inhibit h-amylin amyloid formation using fluorescence detected thioflavin-T binding assays and transmission electron microscopy (TEM). Thioflavin-T is a small dye which is widely used to follow amyloid formation [37, 38]. The in-register, parallel β-sheet structure of amyloid fibrils creates a series of surface grooves that run parallel to the long axis of the fibril and these are believed to form the thioflavin-T binding site. Binding of the dye fixes the position of the benzothiazole and dimethylamino benzyl rings of thioflavin-T and reduces self-quenching, thereby leading to the enhancement of quantum yield observed in the presence of amyloid fibrils [38–41].

**Fig 2:**
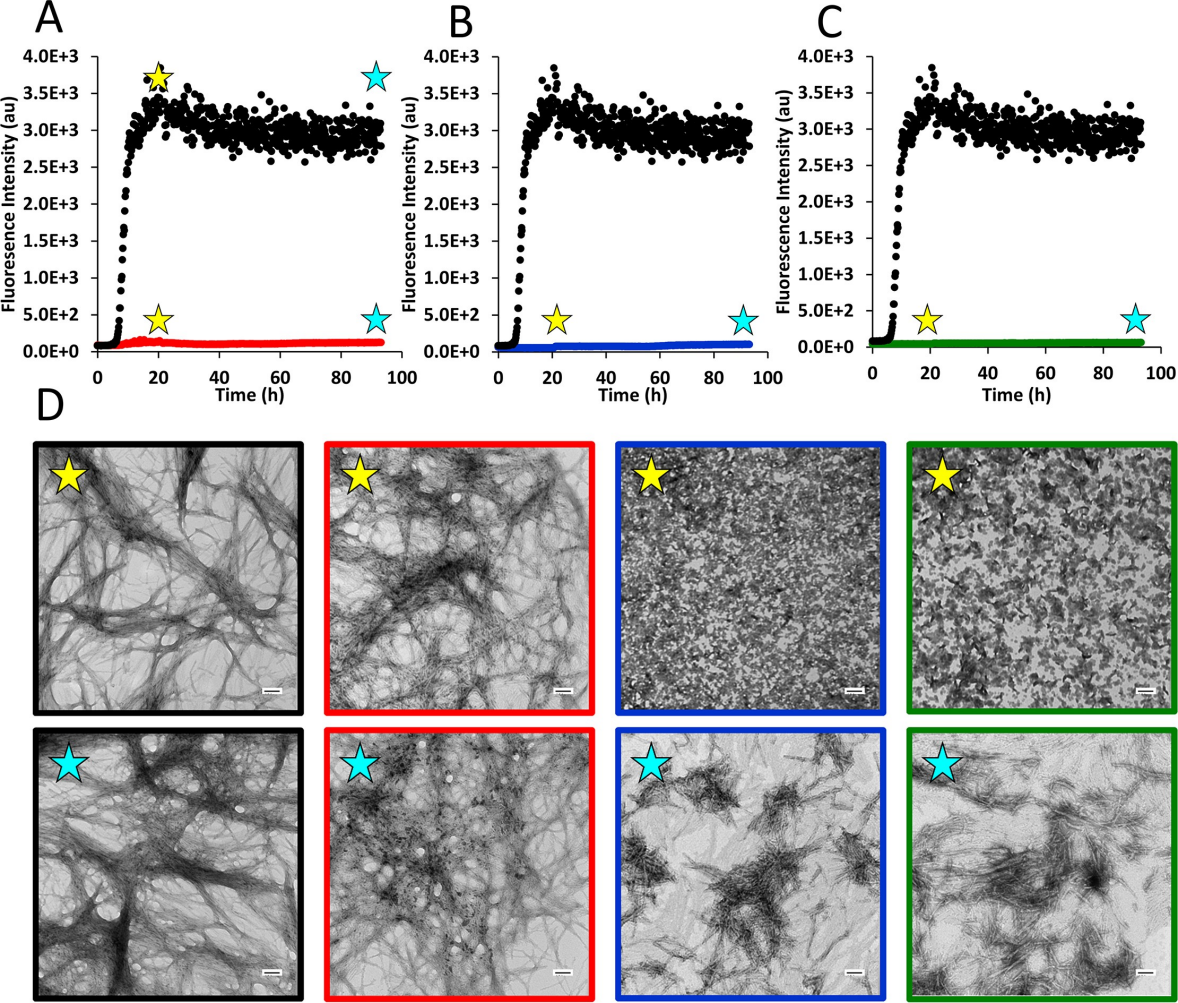
Brilliant blue G (BBG) moderately inhibits h-amylin amyloid formation but interferes with thioflavin-T based assays. Thioflavin-T monitored kinetics assays are shown: (A) h-amylin alone (black) and h-amylin with an equimolar amount of BBG added at the beginning of the experiment (red); (B) h-amylin alone (black); h-amylin with a 5-fold excess of BBG added at the beginning of the experiment (blue) and (C) h-amylin alone (black) and h-amylin with a 10-fold excess amount of BBG added at the beginning of the experiment (green). (D) TEM images were recorded at 20 h (yellow star) and after 93 h (blue star) for all samples: h-amylin alone (black), h-amylin with an equimolar concentration of BBG (red), h-amylin with a 5-fold excess of BBG (blue) and h-amylin with a 10-fold excess amount of BBG (green). Experiments were conducted using 16 μM h-amylin, 32 μM thioflavin-T in 20 mM Tris-HCl with 140 mM KCl at pH 7.4 and 25°C. Scale bars represent 100 nm.

Amyloid formation usually consists of a lag phase followed by a growth/elongation phase in which fibrils grow and/or associate latterly and finally, a final plateau phase. These three phenomenological stages result in a sigmoidal thioflavin-T fluorescence curve. A typical thioflavin-T response is observed for h-amylin in the absence of BBG. Very different results are obtained in the presence of equimolar BBG. In this case, the thioflavin-T signal is greatly diminished and does not change significantly over the time course of the experiment (Fig 2A). The normal interpretation of such a curve would be that the compound is a highly effective amyloid inhibitor. However, TEM studies show that this is not the case. Dense mats of fibrils were observed in the presence and in the absence of BBG at a time corresponding to the plateau region of the thioflavin-t assays (Fig 2D). We repeated these experiments with different samples of BBG and obtained similar results.

ANS has also been used to monitor h-amylin amyloid formation [11], using excitation near 370 nm and monitoring emission near 500 nm. BBG exhibits low absorbance at these wavelengths (S4 Fig) and no significant inner filter effects are expected for the 1:1 and 5:1 BBG to h-amylin samples. In addition, ANS, unlike thioflavin-T, is negatively charged owing to its sulfonate. This may promote stronger interactions with h-amylin amyloid fibrils as h-amylin is positively charged. We examined the effects of BBG at a 1:1, a 5:1 and a 10:1 ratio to h-amylin using ANS based assays. A significant increase in ANS fluorescence is observed in the absence of BBG (S5 Fig). Amyloid fibril formation was confirmed using TEM. In contrast, no significant enhancement in ANS fluorescence is observed when BBG is present, even though the TEM measurements confirm amyloid formation (S5 Fig). The data clearly demonstrates that BBG interferes with ANS based assays of h-amylin amyloid formation. To the best of our knowledge, this is the first demonstration of small molecules interfering with ANS based assays of h-amylin formation. These results show that caution must be used in interpreting ANS based amyloid assays in the absence of confirmatory independent measurements.

### Brilliant blue G inhibits amyloid formation by h-amylin when added in excess

We next examined the consequences of increasing the amount of BBG to a 5-fold and to a 10-fold excess relative to h-amylin. In both cases, no significant thioflavin-T signal is detected in the presence of the compound (Fig 2). TEM analysis of aliquots removed at 20 hours and 93 hours indicate that the compound does have an impact on amyloid formation. The TEM images collected after 20 hours reveal some short fibril like deposits of amyloid fibrils in the sample with 5-fold excess BBG, but fewer and shorter fibrils are visible compared to h-amylin in the absence of the compound. At 93 hours, some fibrils are observed in this sample and those fibrils which are detected appear to be shorter than the ones formed in the absence of BBG (Fig 2). No fibrils are observed at 20 hours for the sample containing a 10-fold excess of the compound, while extensive mats of amyloid fibrils are observed in the TEM images of the sample that contains just h-amylin. At 93 hours, some short fibrils are observed in the sample that contains a 10-fold excess of BBG and these TEM images appear similar to the images collected in the presence of a 5-fold excess of BBG at 93 h (Fig 2D). The physical basis for the reduction in fibril length is not clear, but has been observed with other compounds. The heterogeneous nature of the deposits and the tendency of the fibers to clump together prevents quantitative analysis of the length distribution of the fibers. These experiments demonstrated that BBG is a weak to moderate inhibitor of amylin amyloid formation, but also show that it interferes with thioflavin-T assays. Atomic force microscopy (AFM) confirms that BBG inhibits amyloid formation at high concentrations. We observed amyloid fibrils in samples of h-amylin alone, but only detected modest amount of fibrils, at best, by AFM in the presence of a 10-fold excess BBG (S3 Fig). The changes observed in the presence of high levels of BBG are slightly different using AFM vs TEM. The differences between the AFM results and TEM studies may be due to the washing step required for AFM sample preparation, or may due to the fact that different surfaces are used for AFM and TEM; cleaved mica for AFM and carbon coated copper grids for TEM. The key observation is that, in both cases, a 10-fold excess of BBG has a clear effect upon h-amylin formation.

### Brilliant blue G remodels h-amylin amyloid fibers, but interferes with thioflavin-t based disaggregation assays

**Fig 3:**
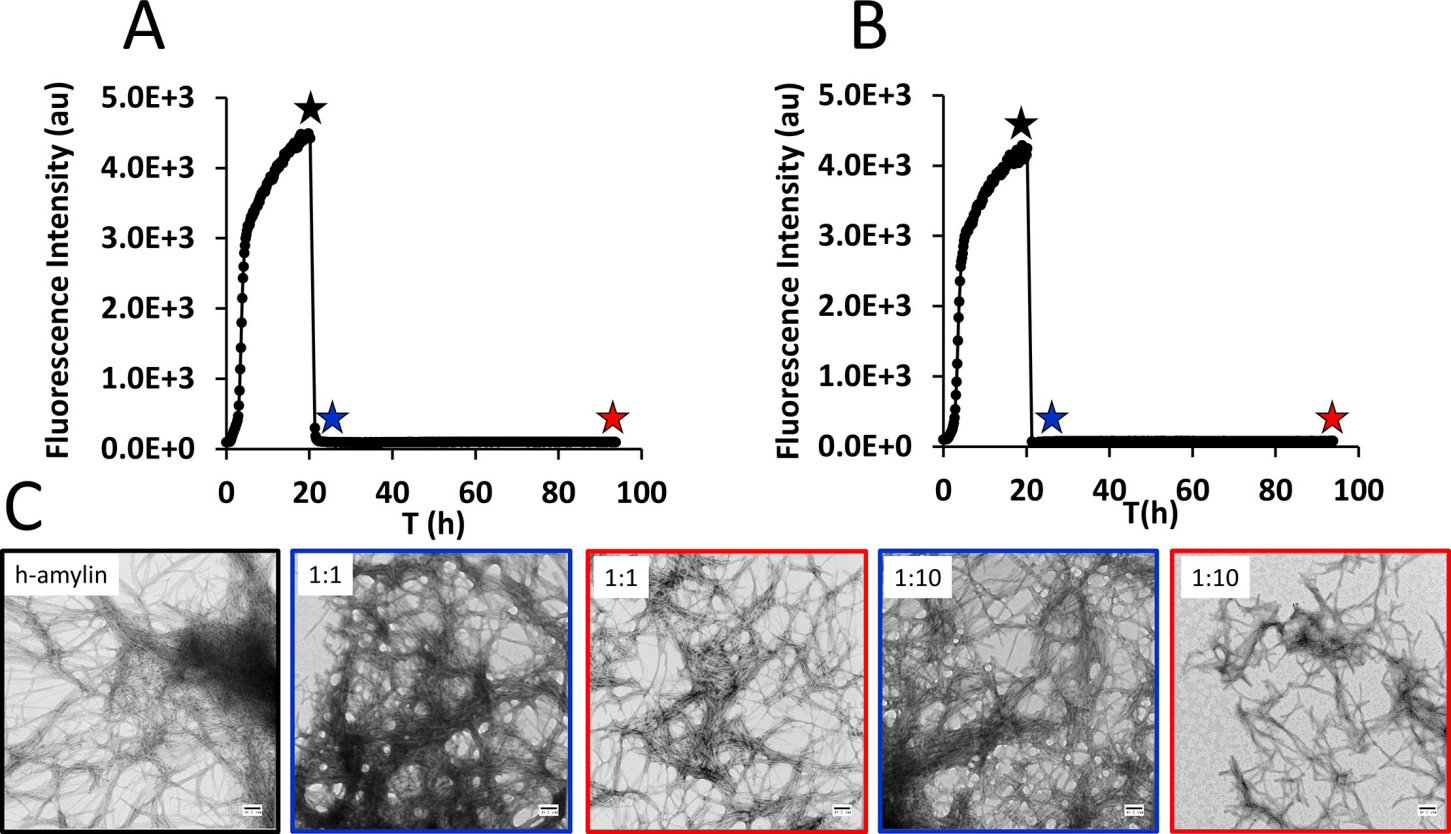
Brilliant blue G remodels h-amylin amyloid fibers, but interferes with amyloid disaggregation assays. Thioflavin-T monitored kinetic curve, h-amylin samples were allowed to form amyloid fibers and (A) an equimolar concentration (based on monomer concentration) and (B) a 10-fold excess concentration (based on monomer concentration) of BBG were added at 20 h. (C) TEM image of a sample collected just prior to the addition of BBG (black star) and TEM images collected at 1 h after addition of BBG (blue star) and at 72 h after addition of BBG (red star). Experiments were conducted with 16 μM h-amylin, 32 μM thioflavin-T in 20 mM Tris-HCl with 140 mM KCl at pH 7.4 and 25°C. Scale bars represent 100 nm.

The experiments described above clearly demonstrate that BBG interferes with thioflavin-T assays if both compounds are added together at the start of an experiment, but also reveal that BBG modulates h-amylin amyloid formation We next examined the effect of adding BBG to pre-formed h-amylin amyloid fibrils. The ability of a compound to disaggregate preformed amyloid fibrils is an important feature of any potential inhibitor since an inhibitor is likely to be clinically administered when some fibrils are already present. Thioflavin-T assays are also a popular tool for following amyloid disassembly *in vitro*. We allowed h-amylin to form amyloid fibrils and confirmed their presence via TEM. BBG was then added at either a 1:1 ratio or a 10:1 ratio of BBG to h-amylin (in monomer units) and the thioflavin-T signal monitored as a function of time (Fig 3A). A rapid decrease in fluorescence was observed immediately after addition of the compound. The normal interpretation of this result would be that the compound is effective at disassembling amyloid fibrils, however TEM images recorded from aliquots that were collected after the thioflavin-T signal had decreased to baseline show that this is not the case. Extensive mats of fibrils were still present in many of the images and they appeared to be similar to the fibrils present before the addition of the dye for the 1:1 sample (Fig 3). These studies show that BBG interferes with thioflavin-T disaggregation assays as well as with amyloid formation assays.

The dye does have an effect on h-amylin amyloid fibrils when added in a 10-fold excess (Fig 3B). A rapid decrease in thioflavin-T fluorescence was observed, as expected when BBG was added. TEM images collected prior to addition of BBG revealed dense mats of amyloid fibrils. TEM images collected after addition of the dye shows that fibrils are still present in some images but appear to be shorter (Fig 3C). Again, the heterogeneous nature of the deposits and their tendency to clump together prevents quantitative analysis of the length distribution of the remodeled samples. AFM imaging confirmed that a 10-fold excess of BBG lead to remodeling of the amyloid fibrils (S6 Fig).

### Brilliant blue G does not significantly protect cultured cells against h-amylin induced toxicity

**Fig 4:**
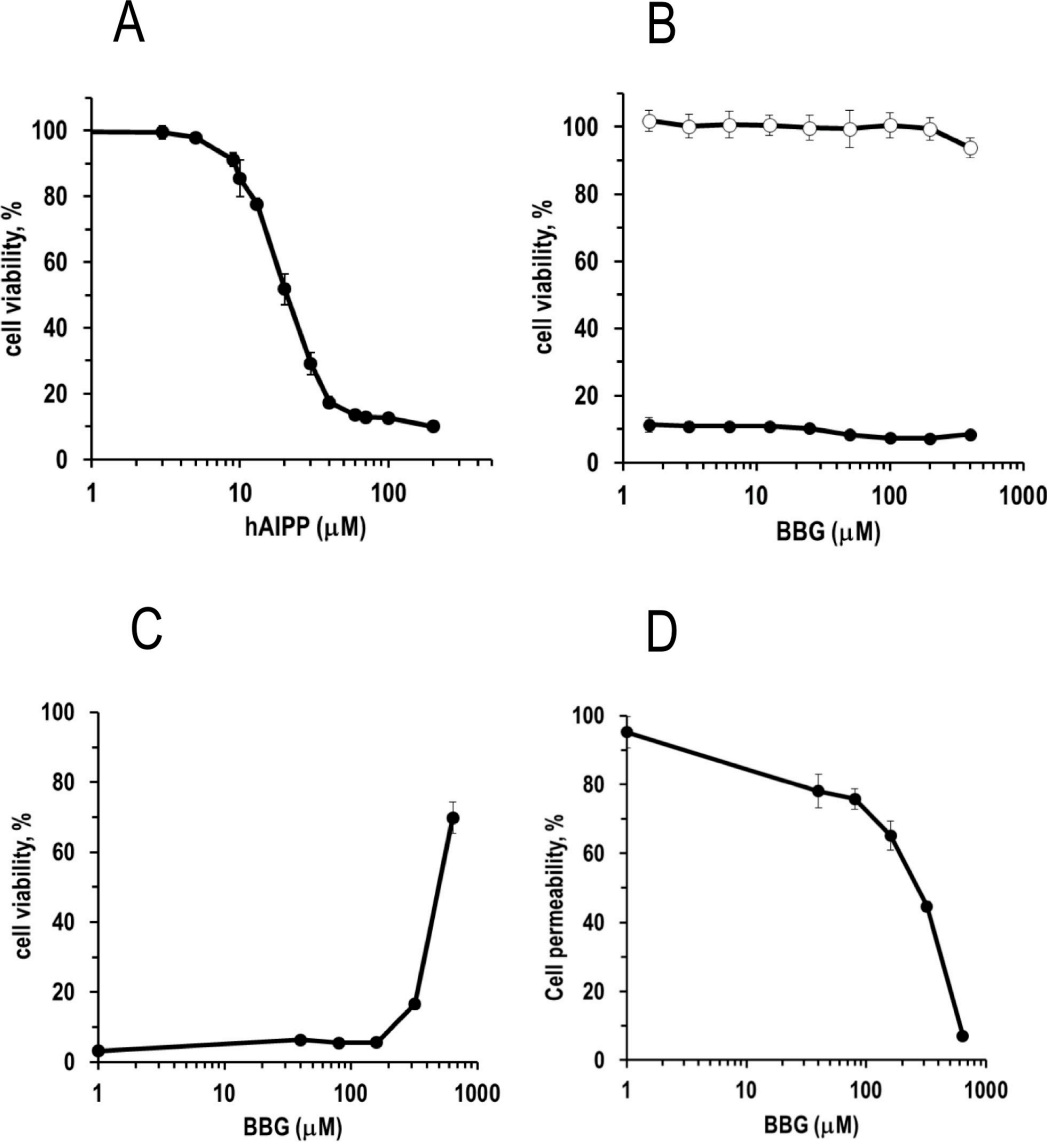
BBG does not efficiently protect cultured β-cells from h-amylin induced toxicity. (A), dose-dependent h-amylin toxicity effects on INS-1 cells as measured by resazurin reduction (Alamar Blue) assays. A concertation of h-amylin corresponding to the estimated concentration of h-amylin required to reduce cell viability by 90%, EC_90_, (40 μM) was used for the BBG cell protection experiments. (B), viability of h-amylin treated (filled circles) and untreated (open circles) INS-1 cells exposed to different concentrations of BBG, as measured by Alamar Blue assays and given as percentage of untreated control (no added amylin). (C), viability of h-amylin treated INS-1 cells exposed to different concentrations of BBG, as measured by the CellTiter-Glo assay and given as percentage of untreated control (no added amylin). (D), plasma membrane integrity of h-amylin treated INS-1 cells exposed to different concentrations of BBG, as measured by the CellTox Green assay and given as percentage of maximal cell permeability. The data from the experiments are plotted as the mean ± SD, n = 3.

BBG has been reported to protect cultured neuroblastoma SH-SY5Y cells from Aβ induced toxicity. We first tested the ability of BBG to protect cultured INS-1 cells and CHO-T cells from h-amylin induced toxicity using Alamar Blue assays. Alamar Blue monitors the integrated redox status of the cells via NAD(P)H-dependent resazurin reduction. We examined the effect of varying amounts of BBG on a concentration of h-amylin, 40 μM, that induced 90% loss in cell viability in the absence of BBG (EC_90_) (Fig 4A). Varying the BBG concentration from 2 to 400 μM had no detectable effect upon cell viability. Even though a 10-fold excess of BBG does have an effect on the self-assembly of amylin into amyloid fibers. It was not possible to conduct experiments at higher BBG concentrations because BBG quenches the fluorescent product produced in the Alamar Blue assay. BBG alone did not exhibit significant toxicity (Fig 4, S7 Fig). Similar results were obtained with both cells lines.

We conducted these assays using two protocols. In one, the cells were incubated for 30 min with BBG in the culture media and then h-amylin was added; in the second, BBG and h-amylin were first mixed and incubated at room temperature for 5 min and then added to the cell culture media. Similar results were obtained in both cases. We also examined the effects of pre-incubating BBG with h-amylin for longer times before applying to cells. h-Amylin in INS-1 medium was incubated at 37°C for 0, 2 and 4 hours and then added to cells. Pre-incubation of 2 and 4 hours lead to greater protection (S8 Fig). h-Amylin aggregation is faster at 37°C than at 25°C and part of the reduction in toxicity may be due to formation of some amyloid fibers as h-amylin fibers have been shown to be non-toxic to cultured cells [11], however the dose dependent BBG effects indicate that the compound does modulate toxicity, albeit weakly.

We next examined the effect of BBG using two other independent cell viability assays. One was an assay that measures net cellular ATP levels via luciferase (CellTiter-Glo 2.0) and the second was an assay that monitors plasma membrane integrity/permeability using a membrane impermeable DNA intercalating fluorescent probe (CellTox Green). These assays are less affected by BBG absorbance and can be used to monitor the effects of BBG at somewhat higher concentrations. Similar results were obtained with these assays and with the Alamar Blue assays. BBG has no detectable effect on cell viability even at a 5-fold excess. At the highest concertation of BBG tested, 600 mM (i.e. at 15-fold excess) partial protection is observed. However, the SAXS data indicates that BBG is unlikely to be monomeric at this concentration.

## Discussion

The data presented here show that BBG inhibits amyloid formation by h-amylin, in the sense that it slows the rate of self-assembly, and it also remodels h-amylin amyloid fibrils when added in excess, although it does not reduce fibrils to monomers. It has been reported that BBG modulates Aβ fiber formation and leads to the formation of amorphous aggregates which are nontoxic to neuronal cells [28, 42]. In contrast, our study shows that the compound fails to protect cultured INS cells or CHO-T from h-amylin toxicity except at high concentrations.

BBG clearly interferes with thioflavin-T and ANS based assays of h-amylin amyloid formation. Small molecules can interfere with thioflavin-T assays by inner filter effects, by acting as a quencher of the fluorescence of bound dye or by competing for the dye binding sites on amyloid fibers or by sequestering thioflavin-T. BBG has no significant absorbance at the excitation or emission wavelength of thioflavin-t, although there is some overlap between the red edge of the dye emission spectra and the blue edge of the BBG absorbance spectrum (S4 Fig). Thus, inner filter effects do not account for the inhibition of the thioflavin-T signal or the ANS signal by BBG [28]. Inner filter effects could interfere with other probes of h-amylin amyloid formation because of spectral overlap. This will be particularly problematic for probes that absorb or emit above 520 nm or in the far ultraviolet as BBG exhibits significant absorbance in these regions in buffer [43] (S4 Fig). BBG has been shown to interfere with the binding of thioflavin-T to amyloid fibrils formed by Aβ. Consequently, we conducted dose dependent studies by adding BBG to a solution of pre form h-amylin amyloid fibers which contained thioflavin-T. Addition of BBG lead to a dose dependent decrease in the fluorescence intensity of a mixture of thioflavin-T and h-amylin amyloid fibrils (S9 Fig) [28]. The EC_50_ for the reduction of thioflavin-T intensity is 6.1 μM BBG for samples containing 16 μM h-amylin and 32 μM thioflavin-T. Given the common mode of binding of thioflavin-T to amyloid fibrils and the similarities between h-amylin and Aβ, BBG is also likely to interfere with the thioflavin-T binding to h-amylin.

**Fig 5:**
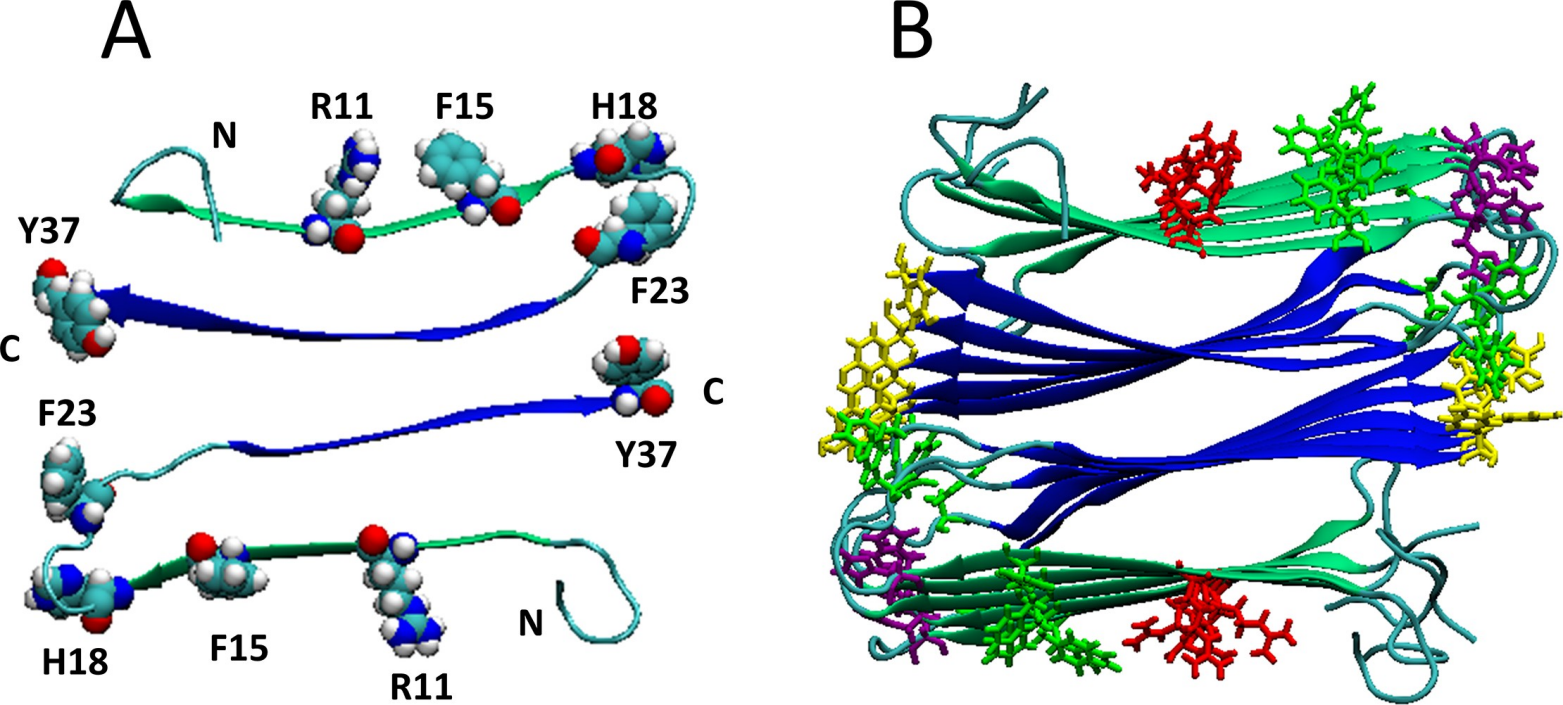
The h-Amylin fibril structure contains patches of exposed aromatic and basic residues. (A) A cross sectional view of the h-amylin fibril with R11, H18 and the three aromatic residues F15, F23, and Y37 shon in space filling presentation. (B) A top down view of the h-amylin fibril model. Five h-amylin molecules are shown per stack. Arg, His and aromatic residues are color coded: Arg-11, Red; His-18, Purple; Phe-15, Green; Phe-23, Green; Tyr-37, Yellow. The structure is based on the solid state NMR model of the amylin amyloid fiber [44].

We conducted sedimentation assays to test if BBG binds to h-amylin amyloid fibrils. h-Amylin fibrils were allowed to form in the presence of BBG, the fibers pelleted and the amount of BBG remaining in the solution was measured by absorbance. Comparison with h-amylin free control samples showed that significant BBG pellets with the fiber. The result is consistent with binding of BBG to h-amylin amyloid fibers (S10 Fig). BBG is believed to interact with basic and aromatic residues. h-Amylin contains three aromatic amino acids Phe-15, Phe-23 and Tyr-37, a single His and single Arg. The parallel in register structure of amyloid fibers will ensure that there are significant aromatic and basic patches on the fiber surface (Fig 5), and these could provide binding sites for BBG.

BBG at high concentrations leads to the remodeling of h-amylin amyloid fibers. A number of compounds have been shown to disaggregate or partially disaggregate h-amylin amyloid fibers, but the mechanism is not understood [45–48]. Possible mechanisms of disaggregation/disassembly include weakening of the fibrils, either by modulation of the structure of the protofibrils by the binding of the small molecule or by inhibiting lateral association of protofibrils. Both of these effects could weaken fibrils and thus promote breakage. A second possibility is that a compound may bind to residual soluble peptide (monomers and oligomers) and thereby disaggregate the fibrils via mass action, i.e. by shifting the equilibrium away from fibrils [49–52]. Of course, both mechanisms could be operative simultaneously. The sedimentation date shows that BBG pellets with IAPP amyloid fibers and indirectly supports the first class of mechanisms as does the fact that BBG appears to displace thioflavin-T from IAPP amyloid fibrils, likely by competing for thioflavin-T binding, however these observations do not rule out effects due to mass action.

This study provides a clear example of small molecule inference with thioflavin-T based assays of h-amylin amyloid formation and also shows that small molecules can interfere with ANS based detection of h-amylin amyloid fibers. These results highlight the importance of confirming dye based assays with complementary methods. The work also highlights the differences between inhibition of Aβ and h-amylin amyloid formation and illustrates the limitations of the approach of using Aβ inhibitors as leads for h-amylin amyloid inhibitors.

## Supporting information

S1 Fig Analysis of the purity and stability of BBG. LC traces are shown for: (a) immediately after dissolving BBG in buffer, the total ion count is plotted vs acquisition time (min), (b) 24 hours after dissolving, the total ion count is plotted vs acquisition time (min), (c) immediately after dissolving BBG in buffer, the absorbance at 610 nm is plotted vs acquisition time (min) and (d) 24 hours after dissolving, the absorbance at 610 nm is plotted vs acquisition time (min).
(TIF)

S2 FigSAXS scattering studies of BBG.X-ray scattering results for 1.0 mM BBG (red) and 0.5 mM BBG (blue). The scattering results were fitted to the correlation length model of Hammouda and coworkers to estimate the radius of gyration [36]. The intensity *I*(*q*) in the correlation length model is given by:
$$I(q)=\frac{A}{q^{n}}+\frac{C}{({q\xi )}^{m}}+B$$
The parameters *A*, *B*, *C*, *n*, *m* and *ξ* are fit to the SAXS data, where *n* and *m* are the Porod and Lorentzian exponents, and *ξ* is the correlation length and gives a measure of the characteristic length scale in the system. The radius of gyration, *R*_*g*_, can be estimated by $R_{g}=\sqrt{2}\xi$.(TIFF)

S3 Fig BBG moderately inhibits h-amylin amyloid formation as judged by AFM measurements. AFM images of (A) h-amylin and (B) h-amylin with a 10-fold excess of BBG added at the beginning of the experiments. AFM images were recorded after 120 h of kinetic assays started.
(TIFF)

S4 Fig BBG dye absorbance spectrum. The spectrum was collected for 32 μM BBG dye in 20 mM Tris-HCl with 140 mM KCl buffer in a 1 cm cell.
(TIF)

S5 Fig BBG interferes with ANS based kinetic assays. ANS monitored kinetic assays are shown: h-amylin alone (black) and h-amylin with (A) an equimolar amount of BBG (red), (B) a 5 fold excess amount of BBG (blue) and (C) a 10 fold excess amount of BBG (green) added at the beginning of the experiment and (D) TEM images were collected at the end of the experiments. Experiments were conducted with 16 μM h-amylin, 4 μM ANS in 20 mM Tris-HCl with 140 mM KCl at 25°C, pH 7.4.
(TIFF)

S6 Fig AFM images show that BBG remodels h-amylin fibers. (A) AFM image of h-amylin fibers just prior to addition of a 10-fold excess of BBG. h-Amylin fiber samples were collected at 20 h after the start of the kinetic assays. (B) AFM image of h-amylin samples 100 h after the addition of BBG to h-amylin fibers.
(TIFF)

S7 Fig BBG does not protect CHO-T cells from h-amylin induced toxicity. 40 μm h-amylin treated cells (filled circles) and untreated (open circles) CHO-T cells shown no change in viability when exposed to different concentrations of BBG, as judged by Alamar blue assays.
(TIF)

S8 Fig Pre incubation of BBG with h-amylin leads to enhanced protection against h-amylin induced toxicity. 40 μM h-amylin in complete INS-1 medium was incubated at 37°C for 0, 2 and 4 hours in the presence of 320 or 640 μM BBG prior to application to INS-1 cells. The cells were further incubated for 24 hours and cell viability and plasma membrane integrity were evaluated by CellTiter-Glo **(A)** and CellTox Green **(B)** assays respectively.
(TIFF)

S9 Fig BBG interferes with thioflavin-T based disaggregation assays. The fluorescence intensity of thioflavin-T bound to h-amylin amyloid fibrils decreases with increasing concentration of BBG. Thioflavin–T fluorescence intensity is normalized to the intensity before the addition of BBG dye. Experiments were conducted at 25°C, pH 7.4, 20 mM Tris-HCl with 140 mM KCl, 32 μM thioflavin-T, 16 μM h-amylin, and various concentrations of BBG. Data were fit to a four-parameter sigmoid curve. The curve has no theoretical significance.
(TIF)

S10 Fig BBG sediments with h-amylin amyloid fibers. 16 μM and 160 μM BBG with and without h-amylin was incubated for 46 hours in 20 mM Tris-HCl with 140 mM KCl buffer at pH 7.4 and samples were centrifuged at 17500 g for an hour and the absorbance of supernatant was measured at 584 nm. (A) BBG and h-amylin at the same concentration. (B) BBG in ten -fold excess to h-amylin. The absorbance of the 160 μM BBG sample without amylin is saturated.
(TIF)
