# Supplementary figures and images for: The triphenylmethane dye brilliant blue G is only moderately effective at inhibiting amyloid formation by human amylin or at disaggregating amylin amyloid fibrils, but interferes with amyloid assays; Implications for inhibitor design
Source: PLoS One. 2019 Aug 12;14(8):e0219130. doi: 10.1371/journal.pone.0219130 (PMC6690547; doi:10.1371/journal.pone.0219130)

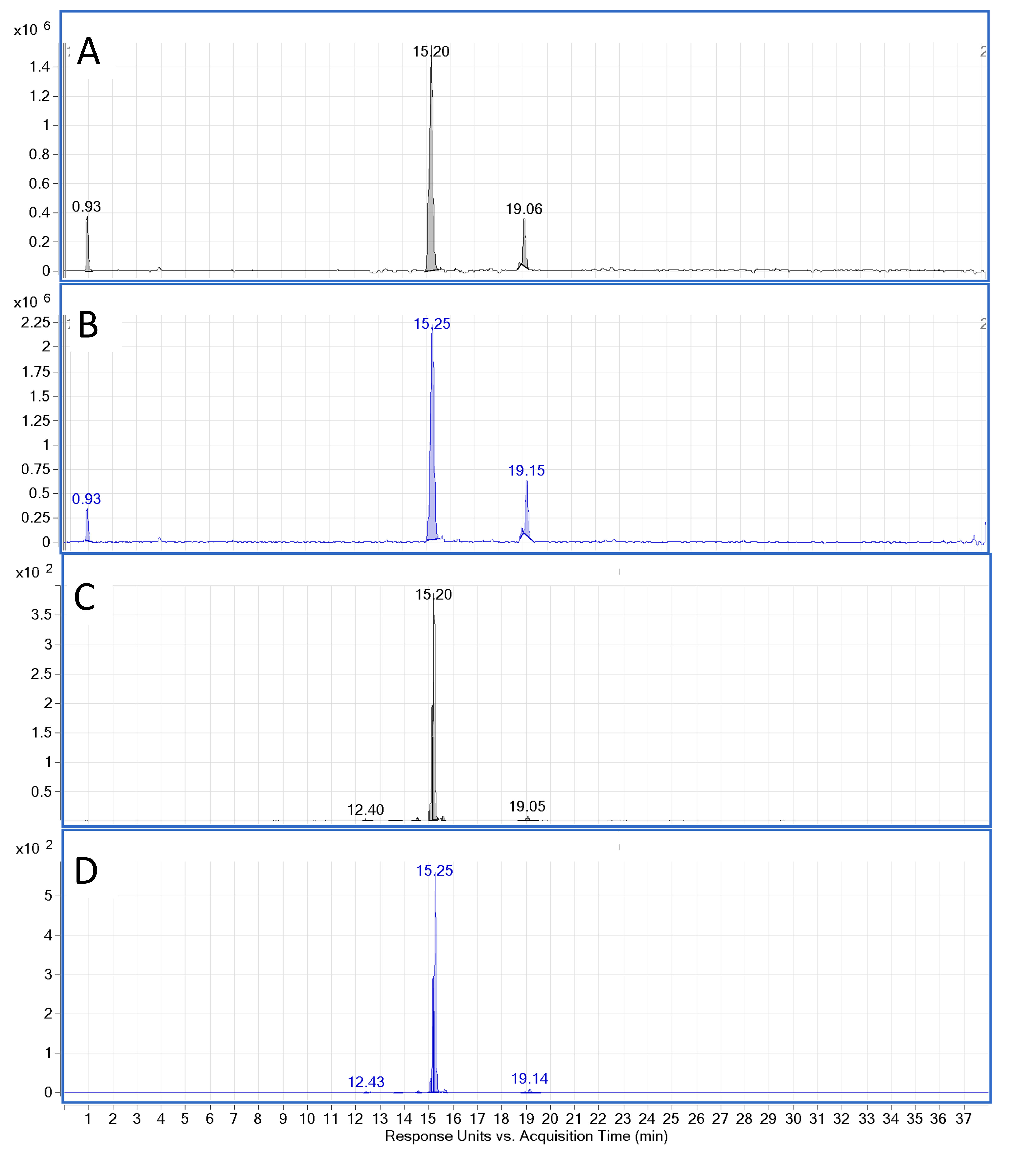

Supplement: S1 Fig — LC traces are shown for: (a) immediately after dissolving BBG in buffer, the total ion count is plotted vs acquisition time (min), (b) 24 hours after dissolving, the total ion count is plotted vs acquisition time (min), (c) immediately after dissolving BBG in buffer, the absorbance at 610 nm is plotted vs acquisition time (min) and (d) 24 hours after dissolving, the absorbance at 610 nm is plotted vs acquisition time (min). (TIF) [file pone.0219130.s001.tif]

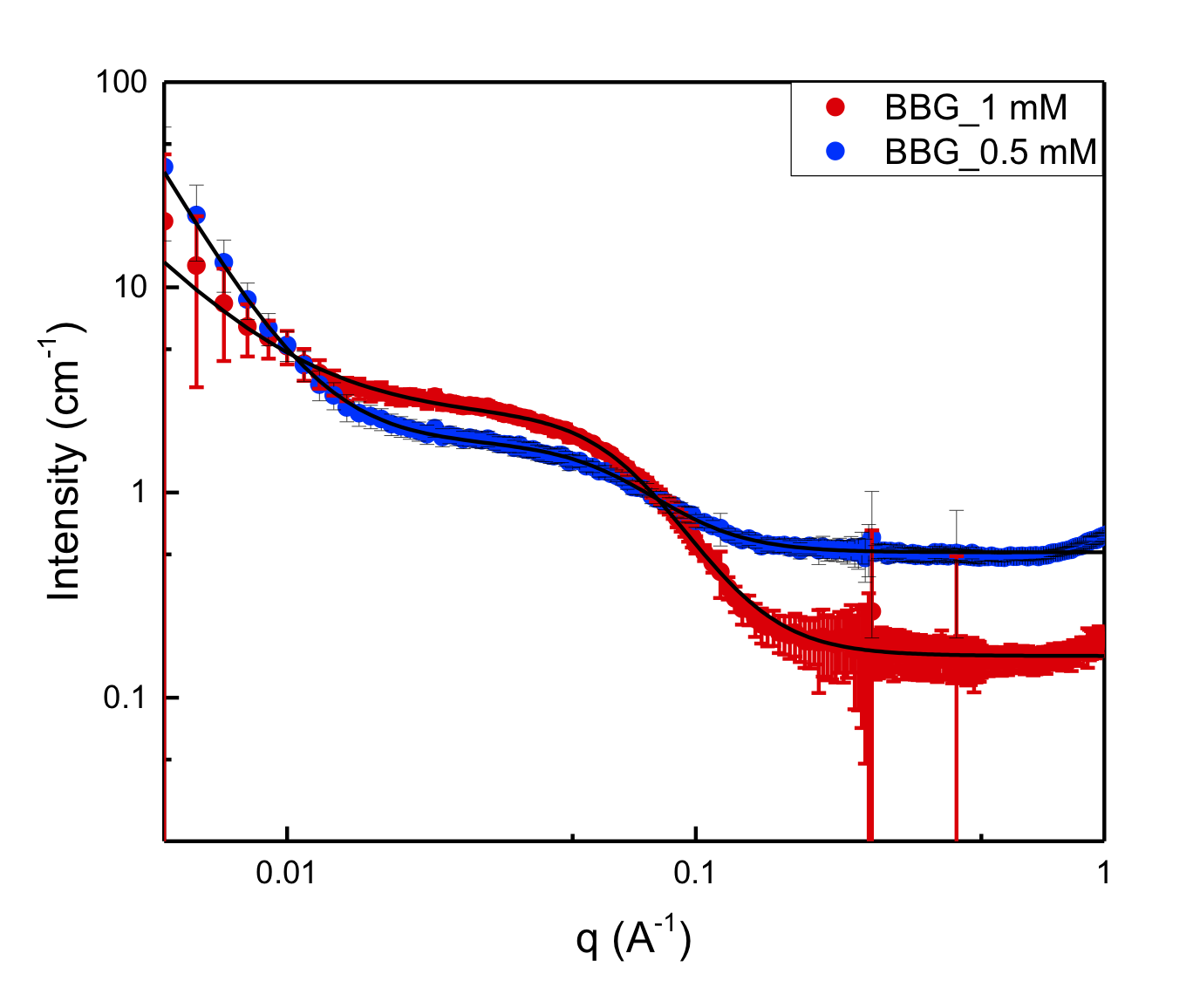

Supplement: S2 Fig — X-ray scattering results for 1.0 mM BBG (red) and 0.5 mM BBG (blue). The scattering results were fitted to the correlation length model of Hammouda and coworkers to estimate the radius of gyration [36]. The intensity I(q) in the correlation length model is given by: I(q)=Aqn+C(qξ)m+B The parameters A, B, C, n, m and ξ are fit to the SAXS data, where n and m are the Porod and Lorentzian exponents, and ξ is the correlation length and gives a measure of the characteristic length scale in the system. The radius of gyration, Rg, can be estimated by Rg=2ξ. (TIFF) [file pone.0219130.s002.tiff]

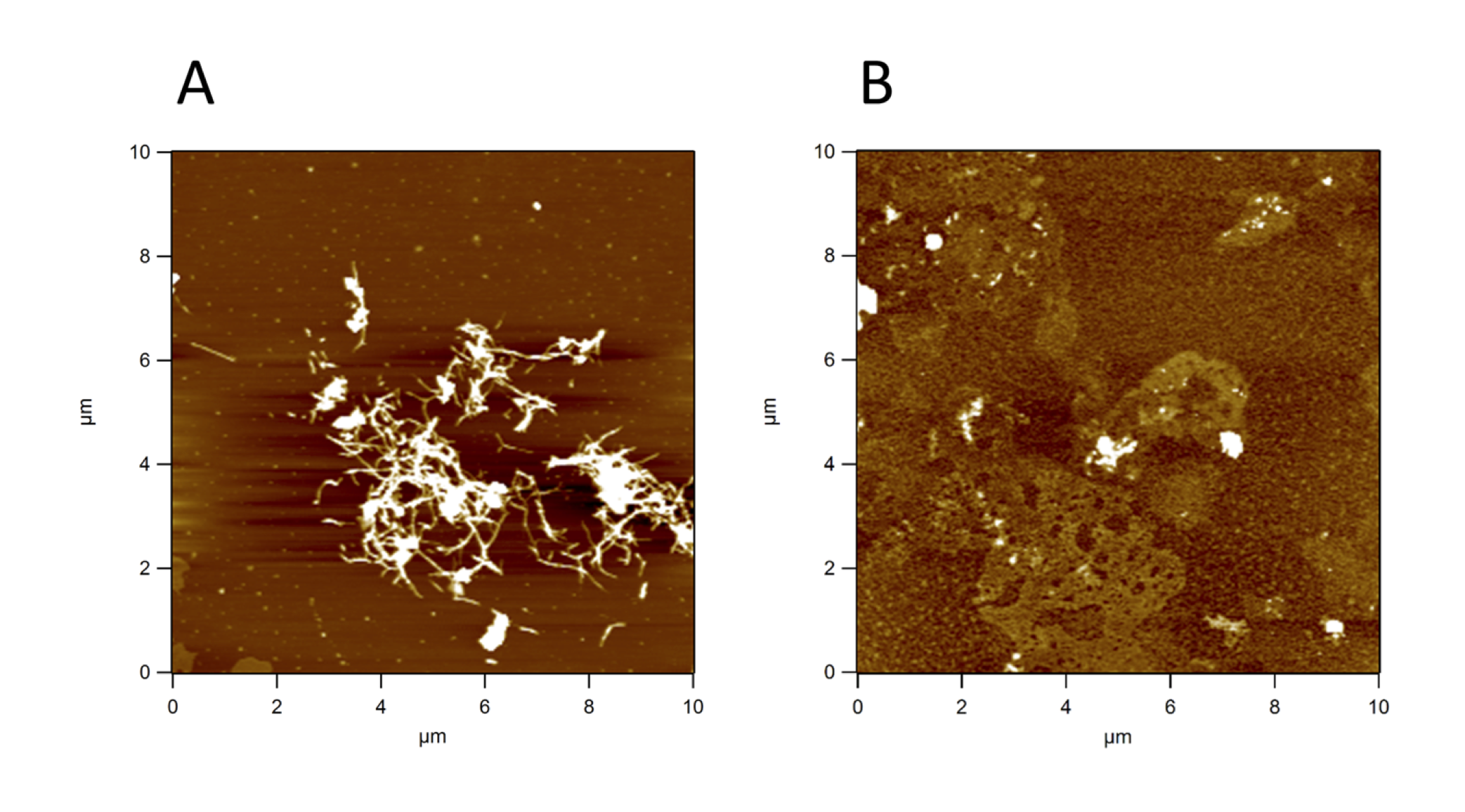

Supplement: S3 Fig — AFM images of (A) h-amylin and (B) h-amylin with a 10-fold excess of BBG added at the beginning of the experiments. AFM images were recorded after 120 h of kinetic assays started. (TIFF) [file pone.0219130.s003.tiff]

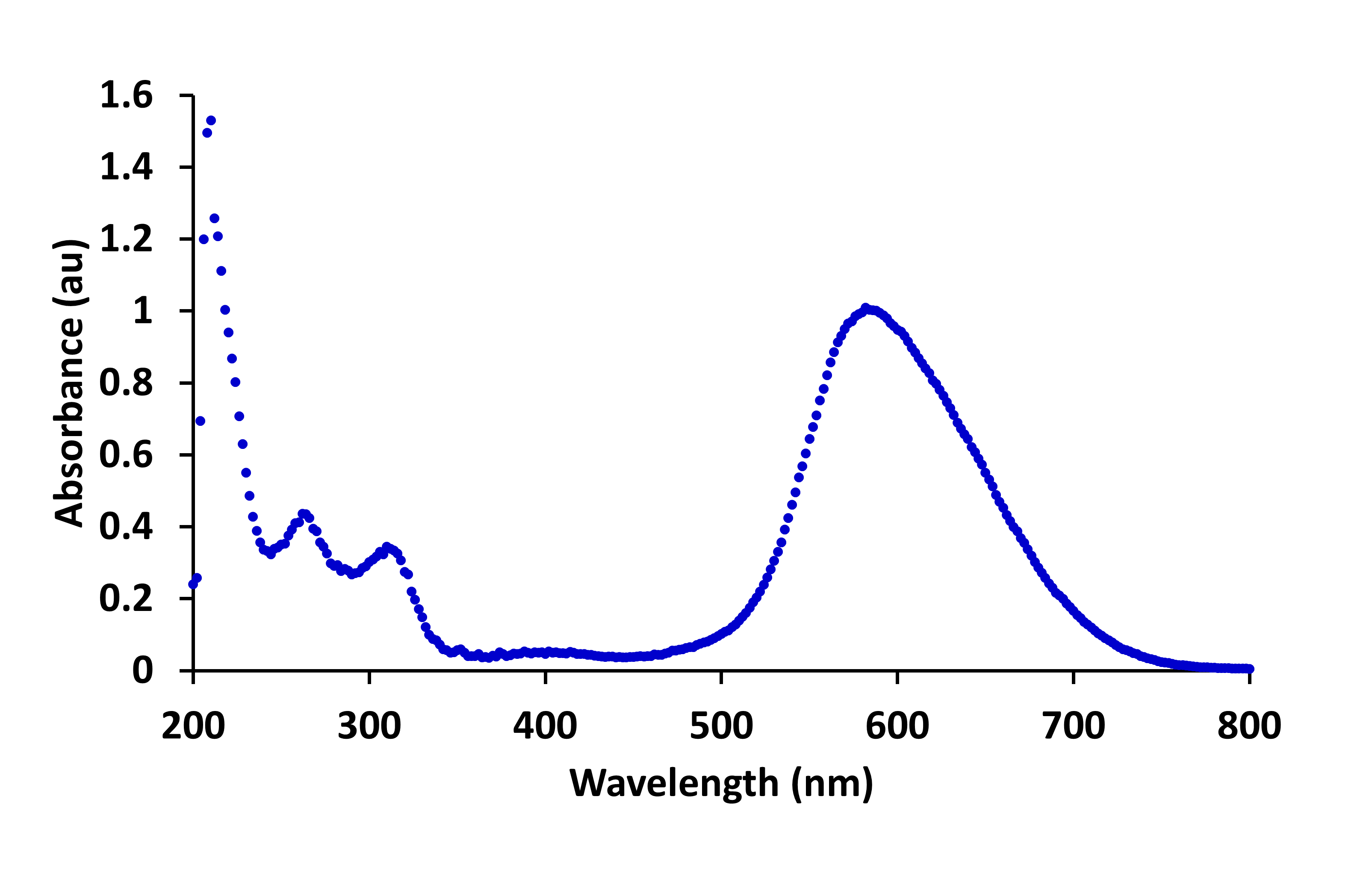

Supplement: S4 Fig — The spectrum was collected for 32 μM BBG dye in 20 mM Tris-HCl with 140 mM KCl buffer in a 1 cm cell. (TIF) [file pone.0219130.s004.tif]

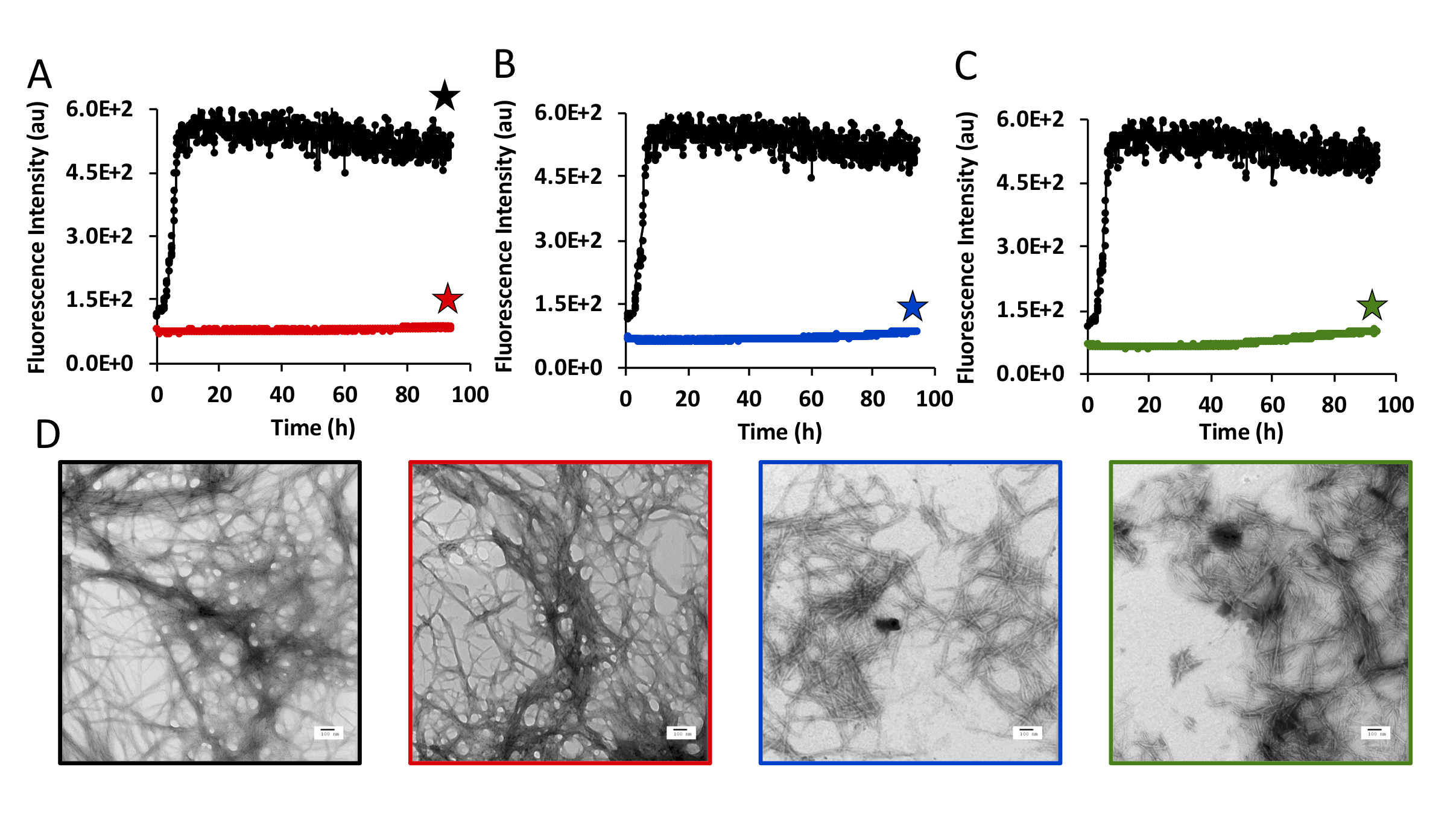

Supplement: S5 Fig — ANS monitored kinetic assays are shown: h-amylin alone (black) and h-amylin with (A) an equimolar amount of BBG (red), (B) a 5 fold excess amount of BBG (blue) and (C) a 10 fold excess amount of BBG (green) added at the beginning of the experiment and (D) TEM images were collected at the end of the experiments. Experiments were conducted with 16 μM h-amylin, 4 μM ANS in 20 mM Tris-HCl with 140 mM KCl at 25°C, pH 7.4. (TIFF) [file pone.0219130.s005.tiff]

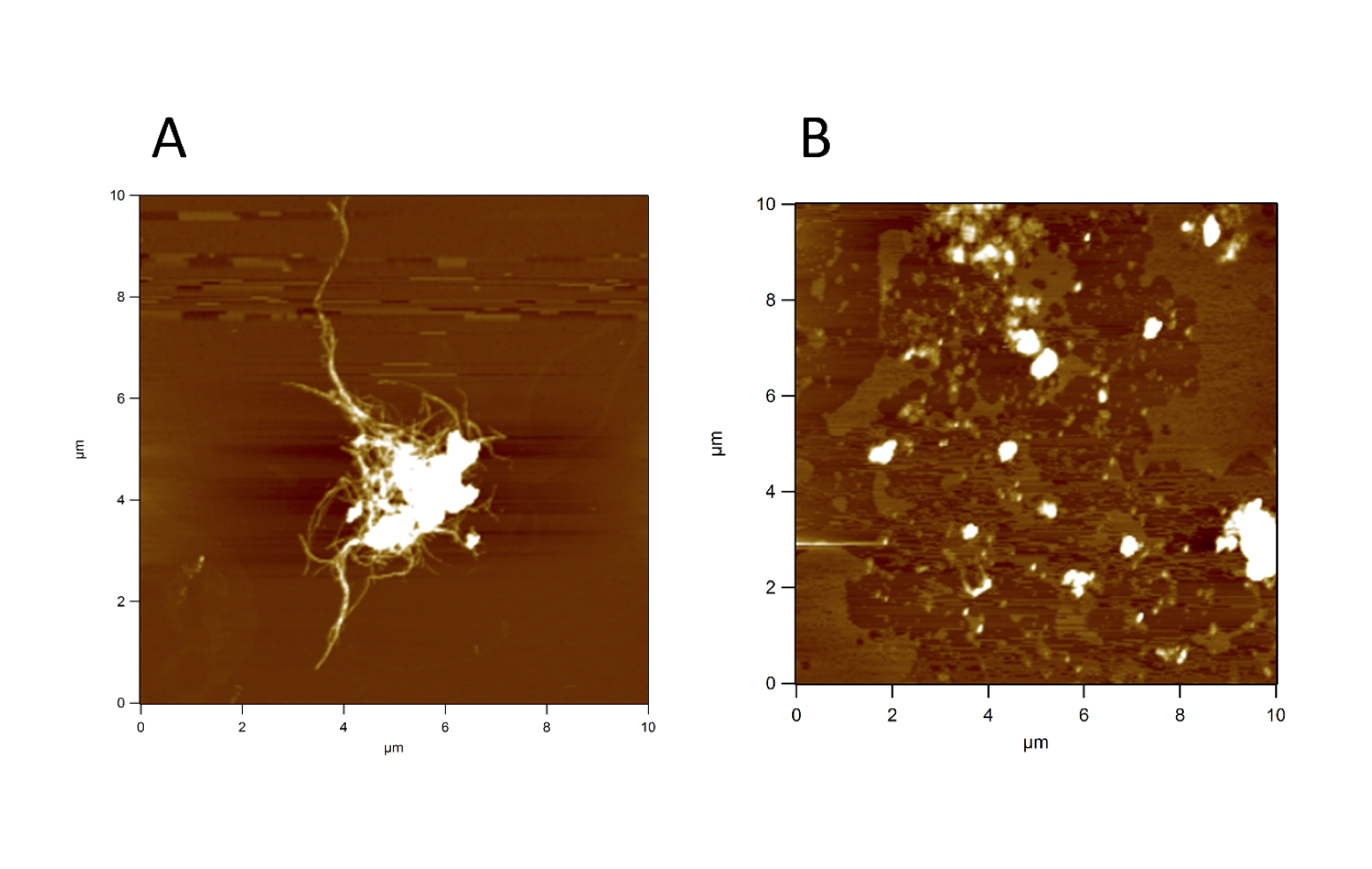

Supplement: S6 Fig — (A) AFM image of h-amylin fibers just prior to addition of a 10-fold excess of BBG. h-Amylin fiber samples were collected at 20 h after the start of the kinetic assays. (B) AFM image of h-amylin samples 100 h after the addition of BBG to h-amylin fibers. (TIFF) [file pone.0219130.s006.tiff]

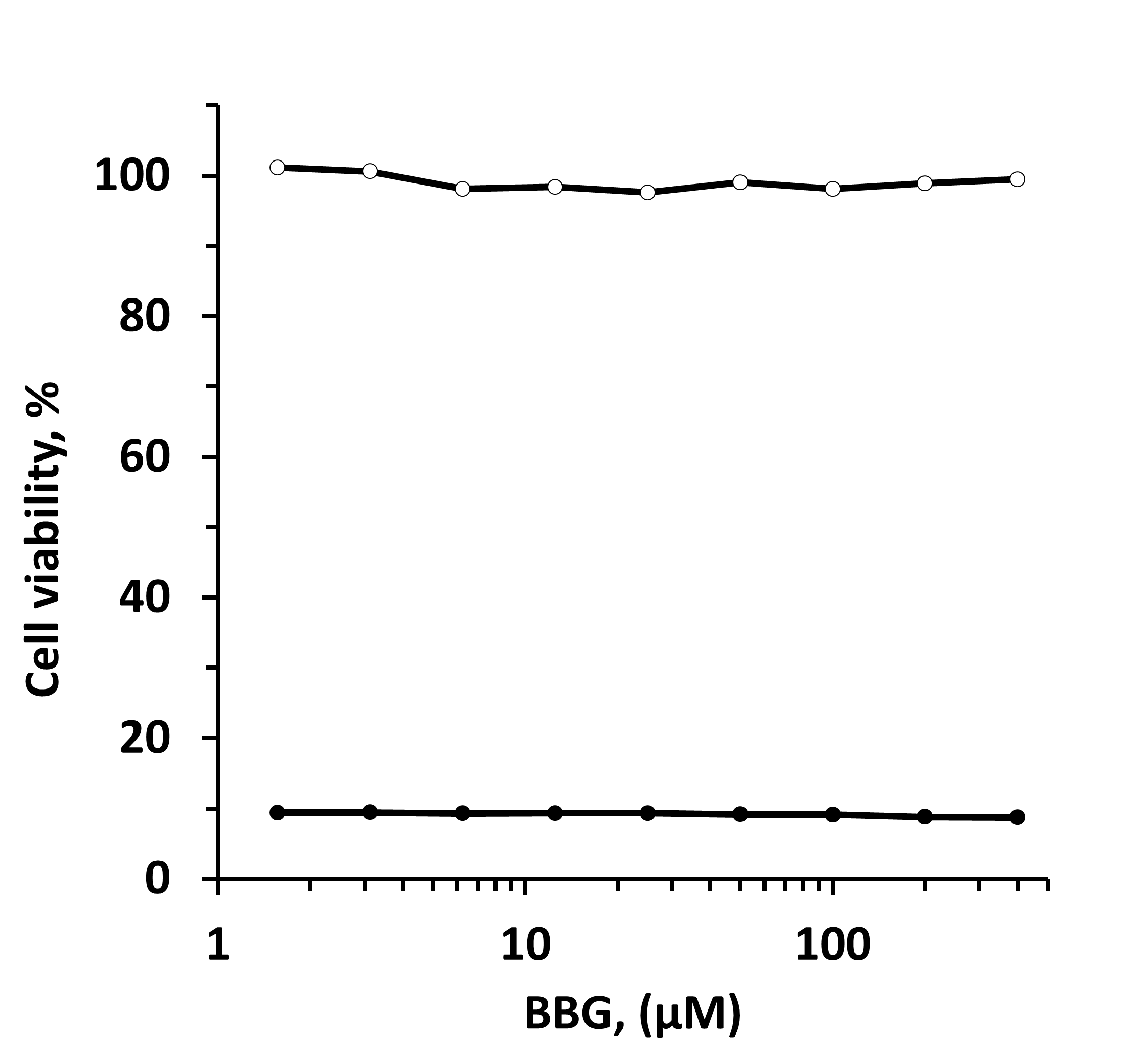

Supplement: S7 Fig — 40 μm h-amylin treated cells (filled circles) and untreated (open circles) CHO-T cells shown no change in viability when exposed to different concentrations of BBG, as judged by Alamar blue assays. (TIF) [file pone.0219130.s007.tif]

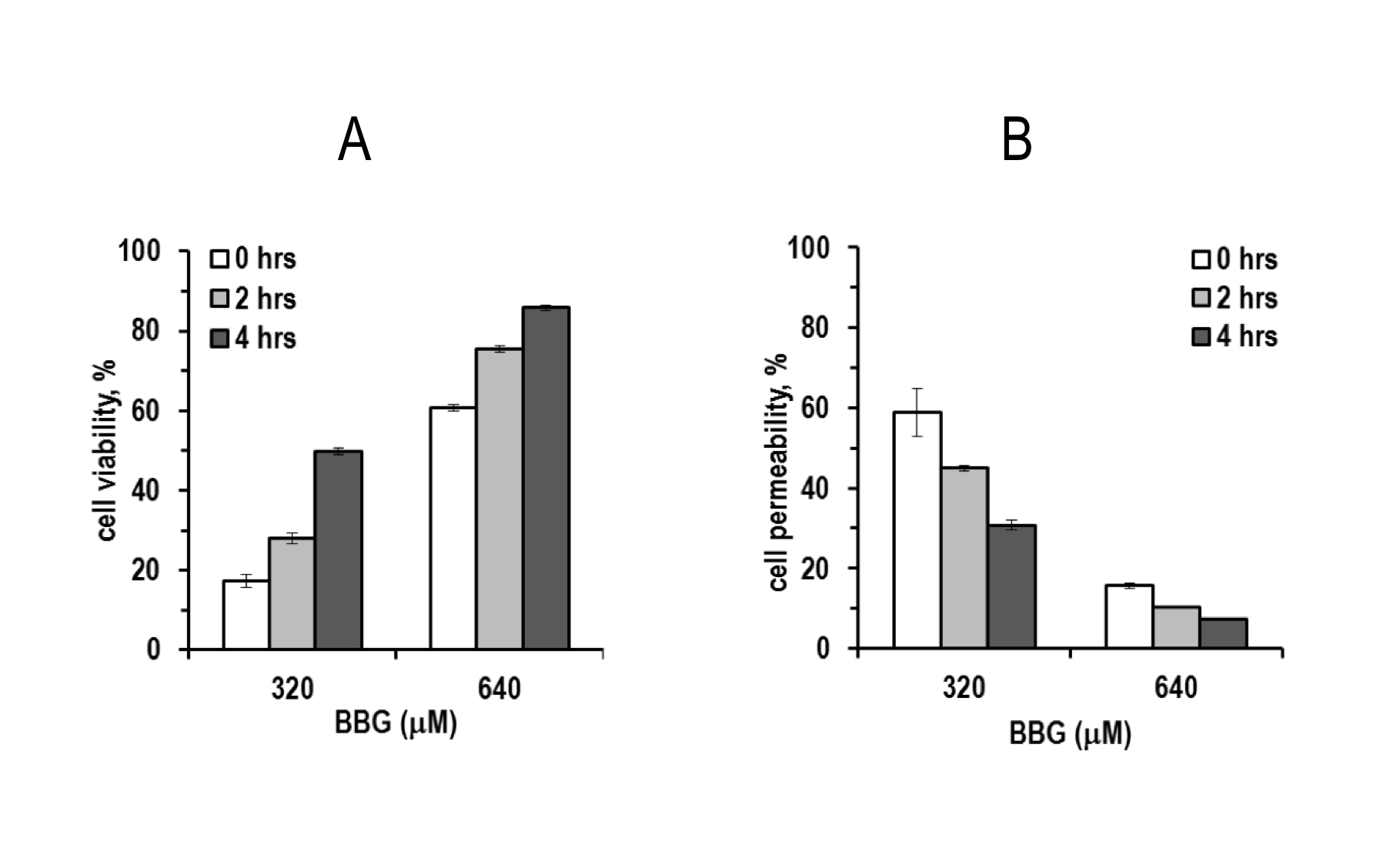

Supplement: S8 Fig — 40 μM h-amylin in complete INS-1 medium was incubated at 37°C for 0, 2 and 4 hours in the presence of 320 or 640 μM BBG prior to application to INS-1 cells. The cells were further incubated for 24 hours and cell viability and plasma membrane integrity were evaluated by CellTiter-Glo (A) and CellTox Green (B) assays respectively. (TIFF) [file pone.0219130.s008.tiff]

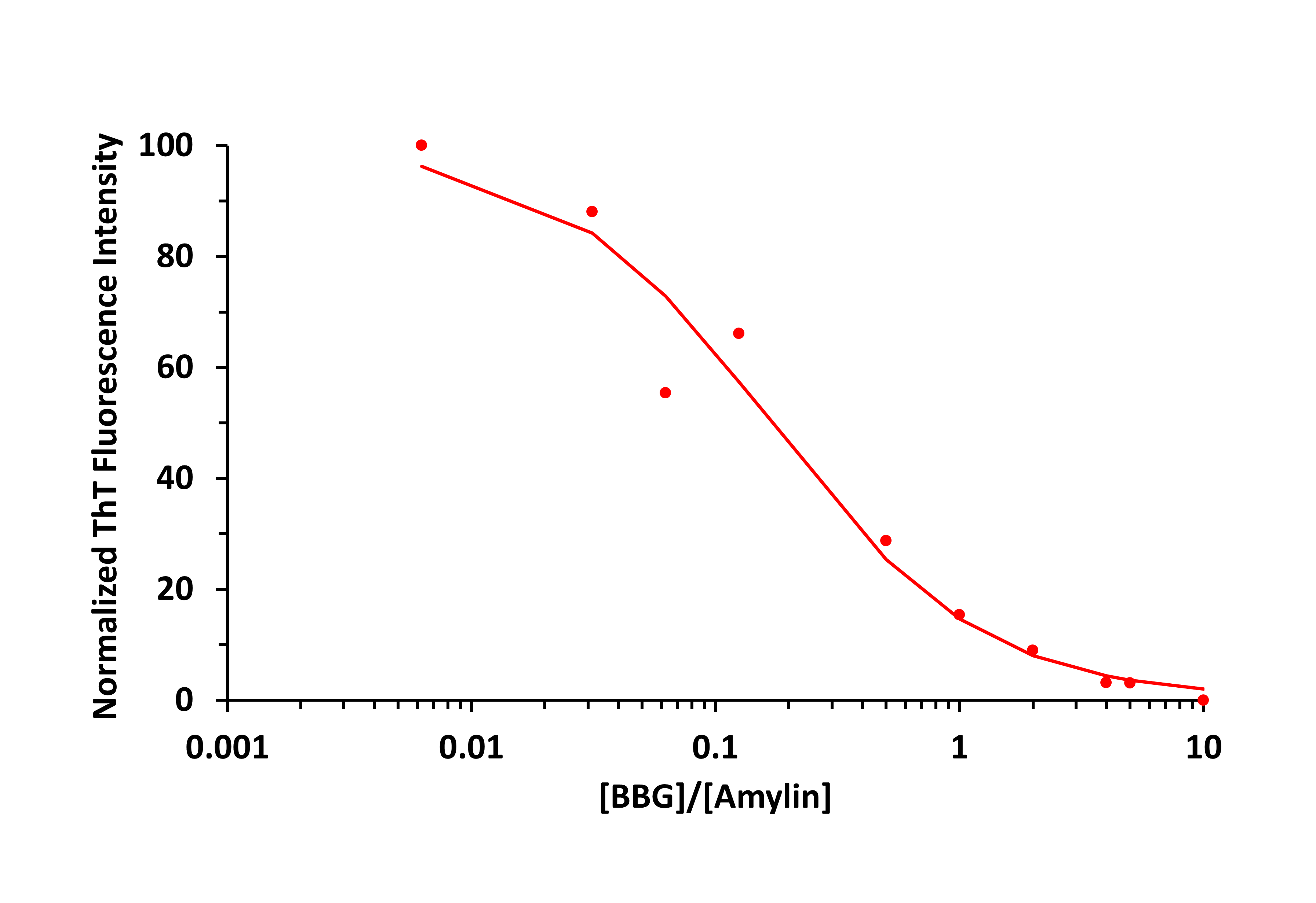

Supplement: S9 Fig — The fluorescence intensity of thioflavin-T bound to h-amylin amyloid fibrils decreases with increasing concentration of BBG. Thioflavin–T fluorescence intensity is normalized to the intensity before the addition of BBG dye. Experiments were conducted at 25°C, pH 7.4, 20 mM Tris-HCl with 140 mM KCl, 32 μM thioflavin-T, 16 μM h-amylin, and various concentrations of BBG. Data were fit to a four-parameter sigmoid curve. The curve has no theoretical significance. (TIF) [file pone.0219130.s009.tif]

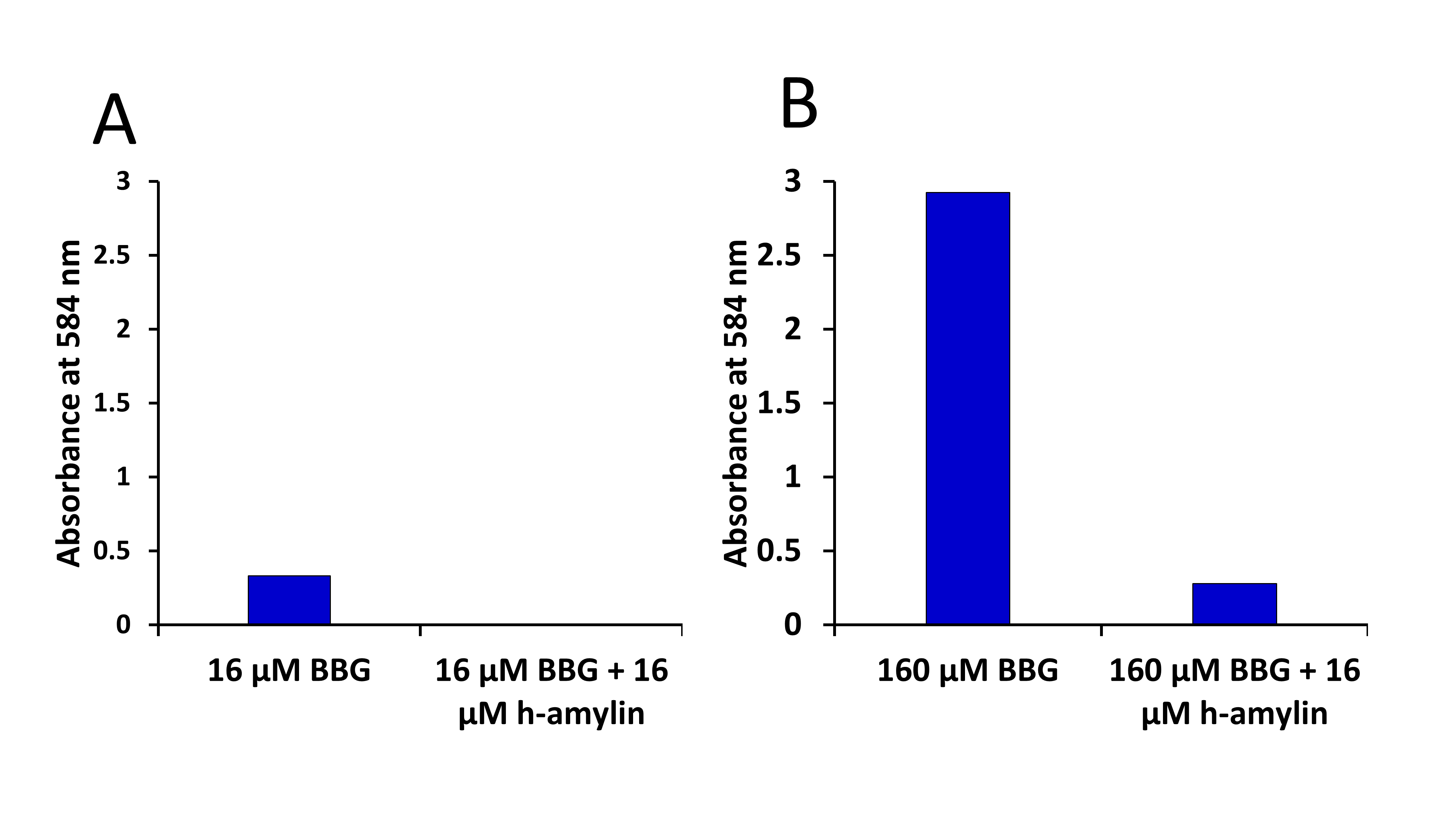

Supplement: S10 Fig — 16 μM and 160 μM BBG with and without h-amylin was incubated for 46 hours in 20 mM Tris-HCl with 140 mM KCl buffer at pH 7.4 and samples were centrifuged at 17500 g for an hour and the absorbance of supernatant was measured at 584 nm. (A) BBG and h-amylin at the same concentration. (B) BBG in ten -fold excess to h-amylin. The absorbance of the 160 μM BBG sample without amylin is saturated. (TIF) [file pone.0219130.s010.tif]
